# Missing value imputation on gene expression data using bee-based algorithm to improve classification performance

**DOI:** 10.1371/journal.pone.0305492

**Published:** 2024-08-29

**Authors:** Kritanat Chungnoy, Tanatorn Tanantong, Pokpong Songmuang

**Affiliations:** 1 Department of Computer Science, Faculty of Science and Technology, Thammasat University (Rangsit Campus), Pathum Thani, Thailand; 2 Thammasat University Research Unit in Data Innovation and Artificial Intelligence, Thammasat University (Rangsit Campus), Pathum Thani, Thailand; Firat Universitesi, TURKEY

## Abstract

Existing missing value imputation methods focused on imputing the data regarding actual values towards a completion of datasets as an input for machine learning tasks. This work proposes an imputation of missing values towards improvement of accuracy performance for classification. The proposed method was based on bee algorithm and the use of k-nearest neighborhood with linear regression to guide on finding the appropriate solution in prevention of randomness. Among the processes, GINI importance score was utilized in selecting values for imputation. The imputed values thus reflected on improving a discriminative power in classification tasks instead of replicating the actual values from the original dataset. In this study, we evaluated the proposed method against frequently used imputation methods such as k-nearest neighborhood, principal components analysis, nonlinear principal, and component analysis to compare root mean square error results and accuracy of using imputed datasets in a classification task. The experimental results indicated that our proposed method obtained the best accuracy results from all datasets comparing to other methods. In comparison to original dataset, the classification model from imputed datasets yielded 15-25% higher accuracy in class prediction. From analysis, the results showed that feature ranking used in a classification process was affected and lead to noticeably change in informativeness as the imputed data from the proposed method played the role to boost a discriminating power.

## Introduction

The missing value issue refers to the presence of missing or incomplete data in a data collection. It occurs when certain observations or variables in a dataset have no recorded values from data collection errors or losing data in data processing. The missing value issue poses challenges in data analysis and machine learning where data are a core input as it can lead to biased results, loss of information, and inaccurate models. Hence, it is important to handle missing values appropriately by employing data imputation techniques.

In biotechnology, DNA microarray technology has created a lot of gene expression data to quantify gene expression levels. [1, 2] Gene expression data analysis has been widely employed in numerous tasks such as biological disciplines, including disease diagnosis, disease prediction, drug design, specific therapy identification [3–8]. Similar to other collected datasets, gene expression data naturally contain missing values. The missing values of a gene expression dataset may come from insufficient resolution, image corruption, fabrication errors, poor hybridization, or contaminants due to dust or scratches on the chip/slide, etc. [9, 10]. Unfortunately, the common data analysis such as classification and clustering techniques rely on a quality of the training dataset as input to maximize the result [8, 9, 11]. Since a process to collect the data is expensive, discarding the data with a missing value is not an option. Thus, handing a missing value issue is essential.

From the studies of related work, missing values in real-world datasets including gene expression datasets are assumed to be missing at random [12, 13]. As data are complex, simple imputation methods, including replacing the missing value by the corresponding row/column average and randomizing the value, tend to result poorly in an analysis task. Hence, complex methods have been applied to help on missing value imputation including weighted k-nearest neighbor (WKNN) [9], local least squares (LLS) [14], multivariate imputation by chained equation (MICE) [15], and Bayesian modeling based on principal component analysis (PCA) [16].

Apparently, these methods consider remaining data values to estimate the missing value. LLS and WKNN select a set of presenting values in the data domain to estimate missing data. For LLS, the more selected values for observation in estimation process, the better value estimation should be relatively obtained. However, it is inefficient to use such a large number of complex data to estimate one missing value from a practical point of view. Furthermore, there is no guarantee that the selected observed data is sufficient to perform imputation well in all cases. For KNN-based imputation, it is suggested to use 10 or 15 similar data for *k*. However, it is reported to perform poorly when k is too small or too large [9]. Thus, its performance depends on the challenge of choosing appropriate sample size and the correlations between presenting data. Another frequently used technique is Principal Component Analysis (PCA) to impute missing data. Unfortunately, it has some drawbacks and limitations. As the method assumes that the data follows a multivariate normal distribution and the missing values are missing at random, it may not be the case in many real-world datasets. When dataset is not accord to the assumptions, bias or distortion in the imputed data may happen. In addition, losing of information or introducing noise in the imputed data may occur in PCA method since it reduces the number of variables and uses expected values instead of observed values. Furthermore, it is unlikely to use for categorical or ordinal variables as it treats them as continuous variables and ignores their discrete nature. Bee algorithm (BA) is also applied to tackle missing data issue. It was reported [31] to outperform other frequent used techniques such as KNN imputation and Genetic algorithm-based imputation method by adding the guideline to modifying the solutions using information gain (IG) score to reduce its randomness in optimization process.

In general, the techniques are designed to fill in missing values in a dataset with values estimated to be as similar as the original values and to improve performance of using the imputed datasets for classification. The most recent works applied advanced techniques such as ensemble method for imputation (ref-Xinshan) by bootstrap sampling for predictions of each method and weighting for producing the final prediction, a technique considering the local similarity structure of missing data using clustering and top K nearest neighbor approaches for imputing the missing value (ref-Aditya), and applying deep learning called psuedo mask imutation (PMI) and GAIN for imputation (ref-Ramon). They focus on filling the missing data of microarray gene expression data, but their work evaluate for imputation accuracy, but does not examine improvement of the performance of a generated model. These works show that their proposed methods have potential to generate missing data that resemble the missing original data. However, it may be more impactful to impute the missing data that may improve accuracy performance of the machine learning during the data imputation by lowering the power to imitate the missing original data in a case of planning to use the generated data for a machine learning-based classification task.

This work purposes a novel method for data imputation that aims to generate the missing data to increase the performance of machine-learning based prediction. The technique is designed based on the bee algorithm and k-nearest neighborhood with linear regression to predict the missing values towards improvement of an accuracy score from a prediction model. Rather than estimating the original values, the proposing technique provides a reliable and completed dataset regarding informativeness of generated values that can help machine learning to classify and predict more accurately. The rest of this paper is organized as follows. Literature review section provides background related to imputation techniques for missing data including statistical and machine learning approach with a brief review of existing works in imputing missing data. Materials and methods section explains the details of the proposed bee-algorithm based imputation method and related materials including datasets and tools used in an experiment. Results section gives experimental results of evaluating imputation error and accuracy from using of imputed data in a classification. Discussion gives analysis of results and finding remarks. Last, Conclusion states contributions of the proposed methods, result summary, and findings in this study.

Contribution of this work

- We proposed a new imputation technique for gene expression data based on bee algorithm with a combination of k-nearest neighbor and linear regression for fitness function and solution generation, call ‘BKL’.
- We demonstrate that the proposed BKL contributes to the improvement of accuracy performance for classification in the task of predicting cancer diseases from gene expression.
- We observed the effect of a classification model generated from the BKL-imputed dataset. We found that ranking of informative features were noticeably shifted as an effect from imputed data. Since the imputed data were generated based on how informativeness they provided, they carried over the significance and cause the shifts of feature ranking resulting in the higher accuracy of classification results.

## Related work

In this section, we describe the concepts and reviews of literature related to the topic of an imputation of missing values. In the review part, we present the summary of literature review in two categories which are statistical imputation method and machine learning imputation method.

### Missing value problem

**Fig 1:**
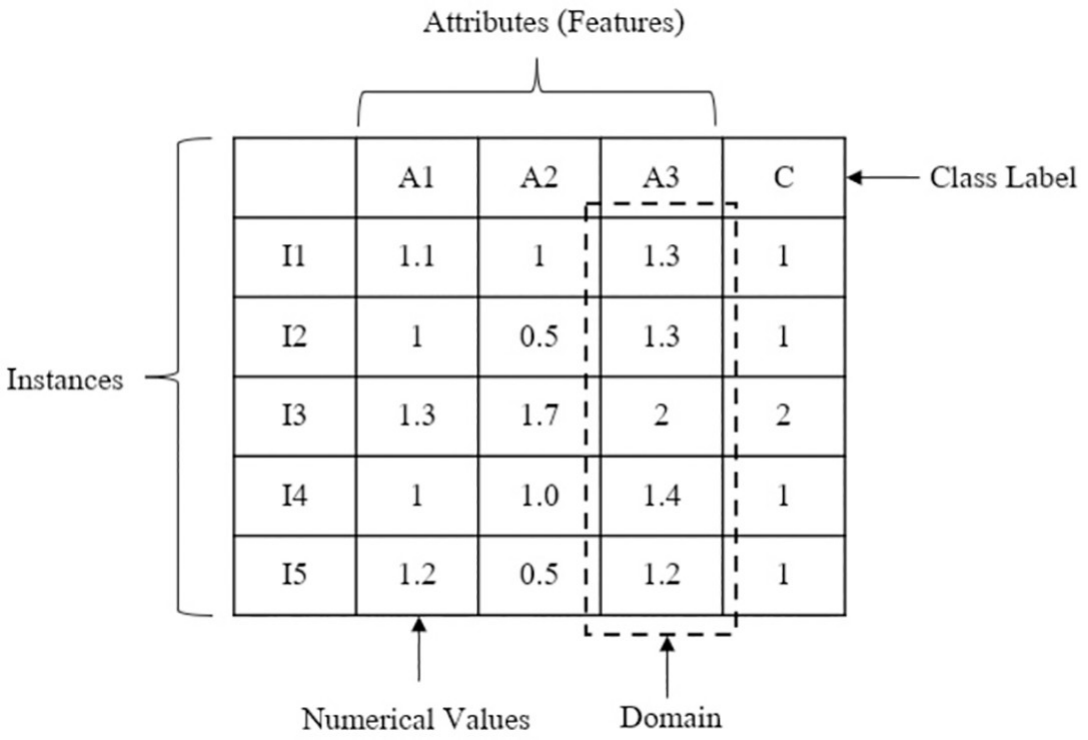
Examples of dataset with missing data and explanations.

Missing value or Missing data is a problem that a value in the dataset is not presented in a designated field [17]. For data mining, missing value is a critical problem that leads to lower accuracy and incorrect conclusion. From the example in Fig 1, missing values (denoted with the symbol ‘?’) are omitted from the dataset. In each attribute, the value can be either categorical, binary, or numerical. The possible values of an attribute are grouped and called a domain.

Missing values a detrimental effect on machine learning models and the overall performance of the learning algorithm, potentially leading to biased results, incorrect imputation, and distorted relationships. Proper handling and imputation techniques are crucial to minimize these effects and ensure reliable model performance.

### Statistical techniques for missing data imputation

In this part, we discuss an approach to use statistical methods for imputing the missing value. This approach is usually applied to impute a single value for each missing position (called single imputation). In some cases, the approach may be customized to impute more than one values call multiple imputations. Famous statistical methods for handling missing values including LLS and PCA with their variation are described below.

#### Principal Component Analysis for imputation

Principal Component Analysis (PCA) imputation is a method used to fill in missing values in a dataset by estimating the missing values based on the relationships between the variables in the dataset. The basic idea behind PCA imputation is to use the principal components of the dataset to estimate the missing values. Principal components are a set of orthogonal vectors that can be used to represent the variation in the data. By projecting the data onto these principal components, we can reduce the dimensionality of the data while retaining most of the variation. To perform PCA imputation, the following steps are typically taken:

- Identify the variables in the dataset that have missing values.
- Compute the principal components of the dataset using the variables that do not have missing values.
- Use the principal components to estimate the missing values in the dataset.
- Repeat steps 2 and 3 several times until the estimated missing values converge to a stable solution.

Nonlinear principal component analysis (NLPCA) is a variation of common PCA by adding a nonlinear generalization to standard principal component analysis (PCA) [18–22]. It generalizes the principal components from straight lines to curves (nonlinear). Thus, the subspace in the original data space which is described by all nonlinear components is also curved. Nonlinear PCA can be achieved by using a neural network [18] with an autoassociative architecture which are also known as autoencoder, replicator network, bottleneck or sandglass type network. Such autoassociative neural network [21] is a multi-layer perceptron that performs an identity mapping, meaning that the output of the network is required to be identical to the input. However, in the middle of the network is a layer that works as a bottleneck in which a reduction of the dimension of the data is enforced. This bottleneck-layer provides the desired component values (scores).

#### Local least squares

Local least squares (LLS) imputation is a method used to fill in missing values in a dataset by statistically estimating the missing values based on the relationships between the variables in the dataset. LLS is an extension of least squares method which selects *k* similar data by L2-norm or Pearson correlation and applies multiple regression to impute missing values [14, 23]for the simple intercept model. The LLS method for data imputation is particularly useful when the dataset contains missing values that are clustered in specific regions or groups. The main concept behind local least squares imputation is to use a local regression model to estimate the missing values. The regression model is fitted using the observed values in a local neighborhood around the missing value. This neighborhood is defined by a set of nearby points that have similar values for the other variables in the dataset. To perform LLS, the following routines are typically taken:

- Identify the variables in the dataset that have missing values.
- For each missing value, define a local neighborhood of nearby points based on the values of the other variables in the dataset.
- Fit a regression model using the observed values in the local neighborhood to estimate the missing value.
- Repeat steps 2 and 3 for all missing values in the dataset.

With the concept, LLS for data imputation is consider showing its best performance when data have a strong local correlation structure. The main challenge of applying LLS for missing data imputation is to assign number of observed data. In Kim et al. [14], the results showed that LLS performed well for a large value of k (over 200). Practically, it is computationally inefficient to use such a large number of complex gene-expression data to only estimate one missing value. Furthermore, there is another report on the performance of LLS in imputation that becomes poorer when k is close to the number of samples. Thus, applying LLS to the task of missing data imputation can be challenging to find appropriate k value in which is different for every dataset.

#### Singular Value Decomposition

Singular Value Decomposition (SVD) imputation is a method used to impute missing values in a dataset based on the low-rank approximation of the data matrix using SVD. SVD is a matrix factorization technique that decomposes a matrix into three constituent matrices: *U*, *Σ*, and *V*. It initializes all missing elements with zero and estimates them as a linear combination of the *k* most significant eigen-variables iteratively until reaches certain convergence threshold [24].

SVD imputation can handle missing values in datasets with arbitrary patterns, including datasets with missing values that are not missing at random, and it preserves the relationships between variables in the dataset while filling in missing values. Unfortunately, SVD is computationally expensive, especially for large datasets, as it involves matrix factorization. Therefore, SVD imputation may not be practical for very large datasets. In addition, SVD assumes that the relationships between variables are linear. In cases of nonlinear relationships in the data, SVD imputation may not provide accurate imputed values. Last, an imputation from SVD only considers the available data matrix without additional information or relationships from other variables or external sources, leading to the limit its effectiveness and potentially lead to suboptimal imputations.

### Machine learning techniques for imputation

Machine learning (ML) for missing data imputation method is a sophisticated procedure. The method used available information from the dataset to estimate the possible value to impute the missing value. Many researchers claimed that the machine learning-based methods are the most suited for the imputation missing values and lead to a significant improvement in prediction accuracy as against imputation based on statistical methods [25]. The techniques to impute missing values are such as K-nearest neighbor imputation (KNN), Genetic Algorithm imputation (GA), and Bees Algorithm imputation (BA).

#### K-nearest neighbor for imputation

The K-nearest neighbor (KNN) method is a common hot deck method by leveraging the values of the nearest neighbors in the dataset. It is a non-parametric imputation technique that can handle both continuous and categorical variables. The K-nearest neighbor vectors are taken from the whole matrix of datasets, except for vectors that have missing values [9]. Vectors then are calculated for similarity measurement such as Euclidean distance [26], and the neighbors are chosen from most similar vectors. To compare the similarity of the metrics, each vector is required to be in the same dimension to estimate the values for imputation [17]. Once the similar vector is obtained, it uses the values of its k nearest neighbors to estimate the missing value for each missing value. For continuous variables, the imputation can be assigned by taking the mean or median of the corresponding neighbors’ values. For categorical variables, the imputation is from selecting the most frequent category as a mode value among the neighbors.

In usage, applied KNN imputation in refinement the missing values in the human activity recognition dataset. As a result, they successfully achieved a complete dataset from filling missing values similar to a pattern of activities as they were in the real dataset. To improve the traditional method, Sanjar et al. [27] proposed a variation called KNN-based most correlated features (KNN-MCF) to use only the most meaningful attributes found using a simulation for the KNN imputation. Their experiment results also signified that the KNN-MCF generates an imputed dataset for a prediction task in which produced prediction model to gain better accuracy than the other method including traditional KNN and statistical methods.

#### Bee algorithm-based imputation

The bee algorithm (BA) is a population-based search algorithm developed by Phan, Ghanbarzadeh et al. in 2005 [28]. It is a nature-inspired optimization algorithm by the behavior of honeybees searching for nectar in a flower field [29, 30]. In applying to solve missing data problem, the setting of food sources is aligned to impute values with the quality of food as a fitness function. The location then becomes a guideline for modifying imputed value in the solution.

Chungnoy et al. presented a method on imputing missing values by applying BA. In their first work [31], they showed that BA performed better in imputation than other techniques such as KNN imputation and GA imputation, but they also reported that most of imputation processes are completely random leading to non-correlated data of the imputation. Therefore, their second work improved the method by adding the guideline to modifying the solutions using information gain (IG) score within the imputation process. Instead of blindly generation, this guideline thus improved the quality of a generated model in terms of relevance and convergence. However, the method still left the values assigning process in random. Later, they proposed the improved version for values assigning process by considering nearest neighbor for heuristic function. The added function is a combination of nearest neighbor and frequency of co-occurrence between observation values and class label instead of randomizing the value. However, the limitation of their methods is that it is only available to the data type of a categorical value.

We have summarized the research related to missing value imputation using statistical and machine learning techniques in the Table 1.

**Table 1:** Summary imputation related works.

Author	Methods	Dataset	Evaluation	Techniques
Scholz, M., & Vigario, R. [18]	NLPCA	EMG Dataset	Classification accuracy	Statistical
Kim H, et.al.	LLS	Gene Expression Data	NRMSE	Statistical
Wei R, et.al	SVD	Metabolomics Datasets	NRMSE	Statistical
Troyanskaya O, et.al.	KNN	Microarray datasets	NRMSE	ML
Sanjar K, et.al	KNN–MCF	House Prices Dataset	Classification accuracy	ML
Chungnoy K, et.al.	BA	Lymphography, Breast cancer,SPECT	NRMSE	ML
Chungnoy K, Songmuang P.	BA	Heart Attack Dataset	NRMSE	ML

## Materials and methods

### Imputation methods

This paper proposes a novel method called Bee algorithm and applies K-Nearest Neighbor-based Linear Regression for missing data imputation (BKL) The objective of this imputation is to fill in missing values in a dataset with estimated data towards the improvement in machine-learning based classification task. Unlike the aim of other methods to replicate the original values, the imputed data which are planned to be used in generating a prediction model will enhance the ability of classification in terms of accuracy.

The core method is based on Bee algorithm (BA) for imputing numerical missing values. The BA part consists of 4 states as scout bees, following bees, solution, and fitness function. Scout bees are tasked to randomly find a solution (imputed value) in the search space and evaluate the fitness of the solutions where they land. The following bees are to follow the scout bees. The following bees then perform a local search to seek further solutions and determine them with fitness function. The following bees become a scout bee and continue to lead following bees. The bees will abandon the solutions if the fitness score is not accepted. Based on original BA, randomness is introduced at several stages including the task of local search to explore the neighborhood of each solution, and selection of solutions based on probabilities proportional to their fitness values. Instead of being random, KNN is used to select a similar instance in a local search task to reduce the search space. The similar instance then is used to create a Linear Regression using the probability-based random important features represented with the calculated GINI importance score.

Objective function in this work is thus to maximize prediction accuracy of a prediction model trained from an imputed dataset. The equation for finding accuracy score of a prediction model is given in (1).

$$\begin{array}{c} \begin{array}{c} Accuracy=\frac{TP+TN}{TP+TN+FP+FN}\times 100 \end{array} \end{array}$$ (1)

Where TP, FN, FP and TN represent the number of true positives, false negatives, false positives and true negatives, respectively.

**Fig 2:**
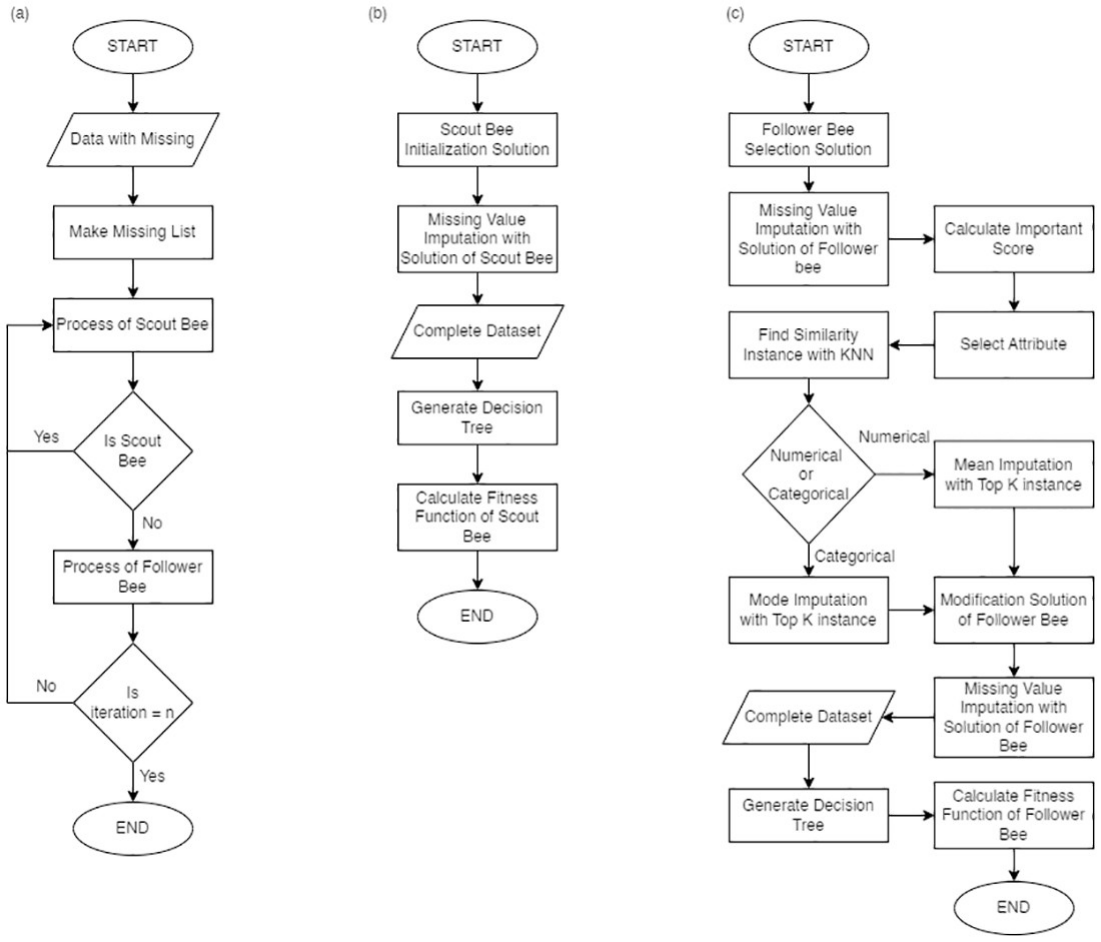
Diagram of BKL imputation method. (a) an overview of the method, (b) processes of scout bee step, and (c) processes of follower bee step.

An overview of BLK is shown in Fig 2a. There are mainly in 3 steps as initial bee, following bee, and scout bee. The pseudo code of all steps is as follows.

1. Initial Step
  (a) Find missing value position in each instance.
  (b) Create a list of missing value position.
2. Scout Bee Step (Fig 2b)
  (a) Randomize the values within a range (min-max) in each feature to n number of scout bees.
  (b) Take the value from each scout bee to impute in the dataset.
  (c) Generate a prediction model of the dataset with imputed data from 2(a) and evaluate the prediction model with the test set for accuracy score and let the accuracy score be a fitness function of a bee to signify a quality of the selected solution. Thus, the higher the fitness function of a solution, a probability for following bees to select the solution.
3. Following Bee Step (Fig 2c)
  (a) Let the m number of Follower bees to random values from scout bees by considering a fitness function to modify a value. The fitness function in this work is an accuracy score of a prediction model calculated by (2). *P*_(*i*, *t*)_ represents probability of choosing a solution, while i refers to *i*^*th*^ following bee, and *t* refers to the total number of all following bees.
  (b) Let a following bee choose a feature in a solution to modify according to a probability from (3) that calculated from GINI importance score using (4) of all features. In (3), *P*_*feature*(*k*)_ is a probability of feature *k*. *k* refers to *k*^*th*^ feature while *m* represents the number of all features, and *l* represents *l*^*th*^ feature. Since the importance score indicates an importance of a feature in classification, it should be focused target in modification for better impact.
  (c) Find top *k* of similar vectors from a set of vectors of the same assigned class using KNN using (5). Within KNN process, only original values in the domain are used in calculation, namely imputed values in the vector are ignored. From (5), *p* is a considered vector, and *q* is a target vector to find similarity. *r* represents *r*^*th*^ feature, and *s* is a number of all features. The obtained 3 linear regression models are then used to predict values for adjustment of imputed value of the said attribute. After similarity scores are calculated, top3 vectors with highest similarity score are created into linear regression model
  (d) Use the adjusted imputed values to replace the imputed values.
  (e) Take dataset with new imputed values (from 3e) to generate a prediction model and evaluate the prediction model with the test set for accuracy score and let the accuracy score be a fitness function of a bee to signify a quality of the selected solution.
4. Repeat 2 and 3 according to the assigned iteration and if iteration > 1, Following bee has a probability to randomly copy imputed values from the previous iteration.
5. END

$$\begin{array}{c} \begin{array}{c} p_{i,t}=\frac{Accuracy}{\sum_{i=1}^{t}Accuracy} \end{array} \end{array}$$ (2)

$$\begin{array}{c} \begin{array}{c} p_{feature(k)}=\frac{GINI\_Score(k)}{\sum_{l=1}^{m}GINI\_Score\left(l\right)} \end{array} \end{array}$$ (3)

$$\begin{array}{c} \begin{array}{c} GINI\_Score\left(k\right)=1-\sum_{n=1}^{o}p_{n}^{2} \end{array} \end{array}$$ (4)

$$\begin{array}{c} \begin{array}{c} d_{pq}=dist\left(x_{p},x_{q}\right)=\sqrt{\sum_{r=1}^{s}{\left(x_{pr}-x_{qr}\right)}^{2}} \end{array} \end{array}$$ (5)

### Main classification method

In this work, a classification was used to evaluate the data imputation. As we focused on the imputation, we adopt decision tree, a supervised classification, to develop a classifier in this study. The Decision Tree model [32] was chosen for its ability to select the locally best attribute to split the dataset on each iteration. In this work, the model was multiclass classification to predict following the given classes from the applied datasets. The parameter settings were as follow Table 2.

**Table 2:** Parameter setting for classification.

Parameter	Values
Criterion	gini
Splitter	best
Maximum depth	None
Minimum samples split	2
Minimum samples leaf	1
Minimum weight fraction leaf	0.0
Maximum features	None
Random state	None
Maximum leaf nodes	None
Minimum impurity decrease	0.0
Class weight	None
Cost-Complexity Pruning alpha	0.0

### Datasets

Data for experiment were gene expression data. The datasets were Breast cancer dataset, Brain Cancer dataset, and Leukemia dataset from Curated Microarray Database [33]. It contained 78 handpicked cancer microarray datasets, extensively curated from 30,000 studies from the Gene Expression Omnibus (GEO), mainly for using in machine learning tasks. The datasets were manually and carefully curated from samples quality, unwanted probes, background correction and normalization, to create a reliable source of data for computational research. As such datasets in practice were valuable, expensive to collect and tentatively contain missing values, we chose these gene expression dataset as our experimental data. For statistics of the selected datasets, the details of dataset including number of instances, attributes and classes are given in Table 3 while class categories are explained in Table 4.

**Table 3:** Datasets for experiments on missing data imputation of numerical type.

Dataset	Training instances	Testing instances	Number of attributes	Number of class
Brain cancer (GSE50161)	103	45	54676	5
Breast cancer (GSE45827)	191	95	54676	6
Leukemia (GSE9476)	187	80	22284	5

**Table 4:** Class variables of the chosen datasets.

Dataset	Classes
Brain cancer(GSE50161)	ependymoma, glioblastoma, medulloblastoma, pilocytic_astrocytoma, normal
Breast cancer (GSE45827)	basal, HER, luminal_B, luminal_A, cell_line, nomal
Leukemia (GSE9476)	AML, Bone_Marrow, PB, PBSC_CD34, Bone_Marrow_CD34

### Data preprocessing

We wanted to investigate how the number of the missing values may affect the imputation and performance of classification. We split the training data and testing data in a ratio of 70:30 following the suggestion from [34–36]. To develop a missing value dataset for experiment, a number of values was randomly removed for 1, 2, 3, 4, 5, and 10—20%. The randomizations were conducted into 5 batches.

### Experiment setting

For evaluation, we imputed the missing values. We wanted to investigate how the number of the missing values affect the imputation and performance of classification. To develop a missing value dataset for experiment, a number of values was randomly removed for 1%, 2%, 3%, 4%, 5%, and 10—90%. The imputed datasets were used to train for classification models and measured for accuracy score from classification results. The data were separated into 5 batches for 5-fold cross-validation in training the classification models. We evaluated the proposed method against 6 frequently used imputation methods including KNN, PPCA. NLPCA, LLS, and SVD. For KNN, 10 and 15 neighbors are chosen. Probabilistic PCA is chosen in this work since the missing values for imputation are all numerical, and [16] indicates that Probabilistic PCA performs better for numerical cases.

Furthermore, we compare the accuracy result against the original data to observe if the imputed data improved a discrimination power of the dataset or not. We also analyzed the imputation performance by Root Mean Squared Error (RMSE) [37] to see how similar the imputed data comparing to the original data. Last, the one-way analysis of variance (ANOVA) is used to determine whether there are any statistically significant differences between accuracy results of classification or not.

### Experiment tool

In this experiment, all experiments were run in a single computer in the same environment as follows.

- Computer Spec
  - Processor: Intel(R) Core(TM) i5-3450 CPU @ 3.10GHz 3.10 GHz
  - RAM DDR3: 32.0 GB
  - Graphic Card: NVIDIA GeForce GTX 1060 6 GB
  - Hard disk: 1 TB
  - System Type: Window 10 64-bit operating system
- Programing language
  - Imputation
    - BKL: Python (3.9.1)
    - MIDASpy: Python (3.9.1) from python MIDASpy library
    - KNN Imputation: Python (3.9.1) with sklearn.impute.KNNImputer library from Scikit-learn
    - PPCA: R, package: pcaMethods
    - NLPCA: R, package: pcaMethods
    - LLS: R, package: pcaMethods
    - SVD: R, package: pcaMethods
  - Classification
    - Decision tree: Python (3.9.1)

## Results

### Classification performance comparing to other imputation methods

We compared the proposed bees-based imputation using combination of K-nearest neighbor and Linear Regression (BKL) against the KNN (KNN-10 for K = 10 and KNN-15 for K = 15), Probabilistic PCA, LLS, SVD, NLPCA and MIDASpy with different missing value percentages on 3 datasets. The imputed data used in classification task and calculated for accuracy as shown in Table 5.

**Table 5:** Classification accuracy result of imputed datasets.

Dataset	Missing (%)	BKL	KNN (K = 10)	KNN (K = 15)	PCA	LLS	SVD	NLPCA	MIDASpy
Brain cancer	1	**96.36**	85.85	87.18	90.40	88.06	87.03	86.82	N/A
	2	**93.59**	86.82	85.69	88.39	86.46	84.46	88.21	N/A
	3	**96.21**	86.26	86.46	86.50	87.21	86.41	85.69	N/A
	4	**93.08**	86.72	86.21	87.10	88.59	87.64	83.33	N/A
	5	**93.74**	85.90	85.49	86.35	87.34	84.36	85.79	N/A
	10	**92.62**	82.51	83.49	85.21	83.32	83.69	84.36	N/A
	20	**92.15**	81.79	84.67	81.69	81.34	81.90	79.49	N/A
	30	**96.42**	80.51	81.85	79.33	N/A	79.43	80.35	N/A
	40	**87.18**	79.18	77.90	76.30	N/A	75.53	75.89	N/A
	50	**89.74**	76.10	76.00	76.82	N/A	78.97	75.07	N/A
	60	**92.31**	70.41	70.97	69.48	N/A	69.48	69.23	N/A
	70	**82.66**	63.38	68.31	64.61	N/A	60.15	59.43	N/A
	80	**79.48**	50.77	46.87	51.33	N/A	53.94	47.28	N/A
	90	N/A	N/A	N/A	N/A	N/A	N/A	N/A	N/A
Breast cancer	1	**87.22**	75.39	74.78	69.08	73.06	75.87	79.57	N/A
	2	**83.83**	74.78	75.43	75.45	74.68	75.13	72.96	N/A
	3	**88.70**	77.74	76.74	78.22	73.11	74.35	73.91	N/A
	4	**85.48**	74.00	73.13	73.64	71.89	71.26	72.43	N/A
	5	**84.39**	72.96	74.04	76.21	75.18	72.96	73.39	N/A
	10	**83.22**	70.91	70.96	71.34	70.94	76.65	71.30	N/A
	20	**85.17**	75.26	76.09	71.96	70.30	72.52	65.61	N/A
	30	**78.26**	61.52	66.79	68.04	N/A	70.95	70.60	N/A
	40	**76.09**	62.09	61.91	67.13	N/A	67.47	61.69	N/A
	50	**73.91**	56.48	53.87	59.95	N/A	63.26	60.08	N/A
	60	**69.57**	52.96	56.13	61.91	N/A	60.04	48.52	N/A
	70	**71.73**	43.43	43.43	49.95	N/A	49.95	44.73	N/A
	80	**63.04**	41.26	42.70	38.26	N/A	32.60	39.60	N/A
	90	N/A	N/A	N/A	N/A	N/A	N/A	N/A	N/A
Leukemia	1	**84.00**	69.30	67.80	68.26	70.67	70.70	67.70	70.00
	2	**84.20**	67.30	66.70	71.65	68.83	68.10	66.90	65.00
	3	**88.10**	69.40	67.80	70.66	67.86	64.90	69.60	67.50
	4	**85.90**	67.90	69.20	71.63	64.84	67.10	68.00	65.99
	5	**87.10**	68.50	68.10	67.96	68.70	71.60	71.30	61.99
	10	**90.50**	67.40	71.00	72.36	66.35	68.10	69.70	66.40
	20	**95.10**	63.10	64.10	70.91	64.76	66.70	71.30	60.00
	30	**88.00**	65.80	67.50	69.10	N/A	70.70	65.60	68.49
	40	**82.00**	63.80	57.60	59.70	N/A	61.90	66.40	63.00
	50	**82.00**	48.70	58.50	54.40	N/A	55.60	53.10	52.00
	60	**82.00**	53.80	46.90	49.10	N/A	49.40	51.20	55.00
	70	**69.00**	37.90	33.40	39.30	N/A	47.40	38.70	45.50
	80	N/A	N/A	N/A	N/A	N/A	N/A	N/A	41.50
	90	N/A	N/A	N/A	N/A	N/A	N/A	N/A	27.00

The results showed that the proposed method yielded better accuracy results from all datasets and missing value percentages. For brain cancer dataset, the accuracy score of BKL was in between 92 to 96% which were apparently higher than the other methods. The accuracy score of the proposed method for leukemia dataset was highest at the 20 and 10 missing value percentage, respectively. This may be interpreted as the imputed values from the proposed method helping to increase a discriminative power of the dataset.

In comparison of the results to advanced techniques, MIDASpy which is a method based on deep learning has a major limitation on imputing missing data in datasets containing large number of features including Brain cancer and Breast cancer as their number of features is enormous, and required large computation resources for imputation. However, MIDASpy was able to impute data for Leukemia dataset for all missing percentage, unlike other methods that could not handle 80-90 percent of missing values. Unfortunately, the classification accuracy of MIDASpy for more than 70% missing values were lower than 50% accuracy and should not be practical to use.

**Fig 3:**
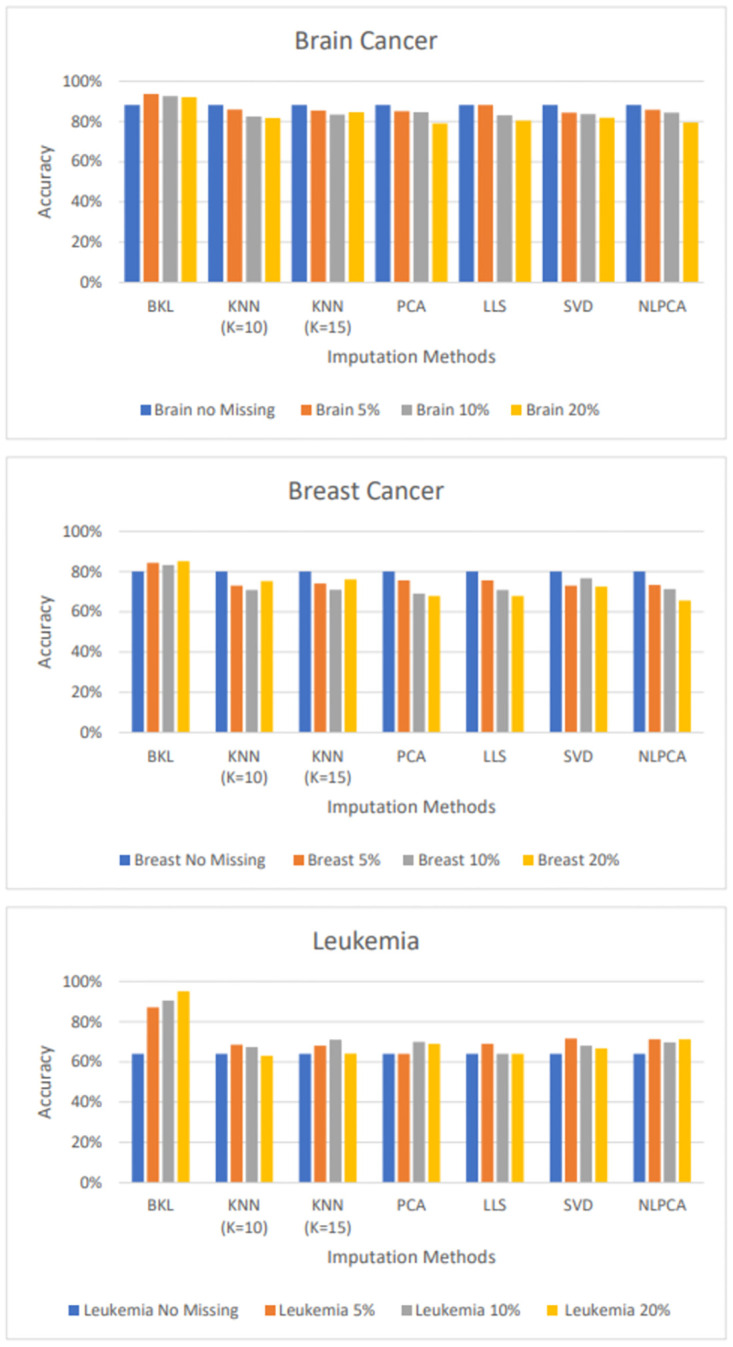
Classification performance of proposing approach comparing to original dataset; 1a result from brain cancer dataset; 1b result from breast cancer dataset; 1c result from leukemia dataset.

Furthermore, we investigated the classification results against the classifier trained from the original dataset as shown in Fig 3. Surprisingly, the classifier models from a dataset with imputed data obtained the higher accuracy score, especially the leukemia datasets which gained 23%, 26%, and 31% higher from 5, 10 and 20% imputed data respectively.

The one-way analysis of variance (ANOVA) was conducted to determine if the difference in accuracy results from each method was significant or not. With Alpha value of 0.05, there was p-value of 1.83 × 10^−12^ which signified that at least one pair among methods have accuracy results with significant difference. Hence, t-Test was conducted to all combinations at Alpha value of 0.0017 and obtained results given in Table 6 which indicated that imputed data from BKL method once were used in a classification task gave significantly better accuracy results than all other methods.

**Table 6:** t-Test results comparing accuracy from using imputed data from different classification methods.

Algorithm	KNN (K = 10)	KNN (K = 15)	PCA	LLS	SVD	NLPCA	MIDASpy
BKL	**7.13 × 10^-9^**	**1.95 × 10^-8^**	**3.77 × 10^-9^**	**0.00011**	**9.54 × 10^-9^**	**5.74 × 10^-9^**	**6.77 × 10^-7^**
KNN (K = 10)		0.94661	0.93122	0.02706	0.71590	0.97871	0.01317
KNN (K = 15)			0.98697	0.03498	0.77307	0.92563	0.01218
PCA				0.02929	0.77657	0.90937	0.01068
LLS					0.05339	0.02498	0.00020
SVD						0.69476	0.00633
NLPCA							0.01369
MIDASpy							

Moreover, we observed how the imputed data affect the classification model and found that rankings of features were noticeably shifted. Since ranking of features directly affected the generated classification model in terms of prioritized features to be considered, these changes in feature rankings should greatly reflect the classification results. We thus selected some for exemplifying the ranking of feature from Leukemia dataset with 5% missing values that were affected by imputation in Table 7.

**Table 7:** Examples of features from Leukemia dataset with 5% missing values that were affected by imputation.

Leukemia 5% Missing				
Feature Order Number	Original Dataset		Imputed dataset from BKL	
	Importance score	Ranking	Importance score	Ranking
feature 2931	0	No Ranking	0.007264	2
feature 4706	0.001504	291	0.000948	428
feature 5549	0.002526	78	0.007311	1
feature 9258	0.00178	251	0.002812	35
feature 9520	0.002693	65	0.000553	563
feature 9554	0.000449	605	0.002165	100
feature 9900	0.002299	138	0.003486	20
feature 11040	0.008067	1	0	No Ranking
feature 13811	0.007239	2	0	No Ranking

### Imputation performance

This evaluation was to observe how the imputed values replicated the original data. A number of values was randomly removed for 1, 2, 3, 4, 5, and 10—90% (excluding the class) to create a dataset with missing values. The RMSE was calculated to show the error rate of the imputation. The higher the error rate, the less accurate the imputed value was. From the results shown in Table 8, the best methods belonged to LLS, KNN and SVD, respectively. The proposed BKL obtained the worst RMSE score amount the competitors. However, since the aim of imputation of the proposed method is to improve classification performance, the higher error mate may reflect that imputing the missing data differently was the key to help to increase a discriminative power for classification.

**Table 8:** RMSE results of imputed datasets.

Dataset	Missing (%)	BKL	KNN (K = 10)	KNN (K = 15)	PCA	LLS	SVD	NLPCA	MIDASpy
Brain cancer	1	2.24	0.53	0.54	0.71	0.47	0.57	0.71	N/A
	2	2.23	0.53	0.54	0.70	0.47	0.56	0.71	N/A
	3	2.24	0.53	0.54	0.71	0.47	0.57	0.71	N/A
	4	2.23	0.53	0.54	0.70	0.47	0.57	0.71	N/A
	5	2.24	0.53	0.54	0.71	0.47	0.57	0.71	N/A
	10	2.23	0.53	0.54	0.70	0.48	0.57	0.70	N/A
	20	2.23	0.54	0.55	0.71	0.49	0.57	0.71	N/A
	30	2.22	0.55	0.56	0.61	N/A	0.61	0.71	N/A
	40	2.21	0.56	0.57	0.61	N/A	0.61	0.71	N/A
	50	2.21	0.57	0.59	0.62	N/A	0.62	0.72	N/A
	60	2.19	0.59	0.61	0.63	N/A	0.63	0.72	N/A
	70	2.18	0.61	0.63	0.64	N/A	0.64	0.72	N/A
	80	2.17	0.65	0.68	0.66	N/A	0.66	0.73	N/A
	90	N/A	N/A	N/A	N/A	N/A	N/A	N/A	N/A
Breast cancer	1	2.25	0.50	0.51	0.66	0.48	0.51	0.61	N/A
	2	2.26	0.50	0.51	0.61	0.48	0.51	0.61	N/A
	3	2.25	0.50	0.51	0.61	0.48	0.51	0.61	N/A
	4	2.25	0.50	0.51	0.61	0.48	0.51	0.61	N/A
	5	2.25	0.50	0.51	0.61	0.48	0.51	0.61	N/A
	10	2.25	0.50	0.51	0.61	0.48	0.51	0.61	N/A
	20	2.24	0.51	0.52	0.61	0.49	0.52	0.61	N/A
	30	2.23	0.47	0.47	0.49	N/A	0.49	0.56	N/A
	40	2.23	0.47	0.48	0.49	N/A	0.49	0.56	N/A
	50	2.22	0.49	0.50	0.51	N/A	0.51	0.57	N/A
	60	2.21	0.50	0.51	0.52	N/A	0.52	0.58	N/A
	70	2.19	0.53	0.54	0.54	N/A	0.54	0.59	N/A
	80	2.19	0.55	0.57	0.56	N/A	0.60	0.60	N/A
	90	N/A	N/A	N/A	N/A	N/A	N/A	N/A	N/A
Leukemia	1	2.08	0.32	0.34	0.45	0.28	0.32	0.45	1.33
	2	2.09	0.33	0.35	0.46	0.29	0.33	0.46	1.43
	3	2.08	0.33	0.35	0.46	0.28	0.33	0.46	1.31
	4	2.08	0.33	0.35	0.46	0.28	0.33	0.46	1.38
	5	2.08	0.33	0.35	0.46	0.28	0.33	0.46	1.25
	10	2.08	0.33	0.35	0.46	0.29	0.33	0.46	1.36
	20	2.08	0.34	0.36	0.46	0.29	0.33	0.46	1.34
	30	2.08	0.35	0.37	0.36	N/A	0.36	0.46	1.36
	40	2.08	0.36	0.38	0.37	N/A	0.37	0.47	1.26
	50	2.07	0.38	0.40	0.38	N/A	0.37	0.47	1.26
	60	2.07	0.39	0.42	0.39	N/A	0.38	0.47	1.44
	70	2.07	0.42	0.44	0.41	N/A	0.41	0.47	1.50
	80	N/A	N/A	N/A	N/A	N/A	N/A	N/A	1.45
	90	N/A	N/A	N/A	N/A	N/A	N/A	N/A	1.86

## Discussion

For traditional imputation methods, the accuracy scores were lower than or equal to the classifier generated from the original data (no missing value). The results were aligned to [38], who studied the effects of imputing data using KNN, LLS and BPCA on the classification performance and found that the imputation did not affect classification performance. Thus, it could be concluded that these imputation methods did not have any benefit on classification, except to fill the missing data for training. However, the classifier results in terms of accuracy from imputed dataset by BKL were apparently better than those from the original data as shown in Fig 2a, 2b and 2c.

The experiment results clearly showed that BKL had the potential in imputing missing values of gene expression data. Based on results from Table 8, the method may not impute what considered accurate to the original data as it yielded around 2.07 to 2.26 RMSE, which were worse than other competing methods. This indicates that the generated data from the BKL were not exactly the same as the original data. Instead, BKL tentatively generated the different data that had an impact to improve a discriminative power of the dataset which was reflected in the accuracy score from the classification task (Table 5). Thus, the generated data from the proposed technique may be noticeably different to the original data but more impactful towards the later classification task since the main aim is to improve accuracy result of the classification instead of imitating the original data. As the method involved in applying KNN and LR based on features with the GINI importance score, the imputation prioritized important features over accurate original data. The values selected based on the important features hence were more informative than the original data in terms of a discriminative power to a classification model. As exemplified in Table 7, the feature rankings were changed, and some features that were ranked lowly with the original data were more prioritized for the imputed dataset. This led to emphasize the features that had an implicit discriminative power to boost a classification result. The t-Test confirmed that the imputed data generated by the proposed method exhibited significantly higher accuracy compared to other imputation methods, making it an excellent choice as imputed dataset used in classification tasks.

Regarding missing value percentage, the experimental results were different for the testing datasets. The performance on leukemia dataset indicated that the more missing value for imputation, the better the classification performed. On the other hands, the performances of classification were similar for the brain cancer and breast cancer dataset regardless of an imputation amount. The results indicated that some features of the leukemia dataset were more implicitly significant than other, and the importance score boosted their significance to become more prioritized in a classification mode and increased the classification performance.

In terms of usage, although BKL may show great impact towards performance improvement of the classification of missing value datasets, the method was not designed to replicate the actual missing values, it may not be suitable for tasks that require actual data for analysis such as gene modeling and gene variation analysis, which needs accurate data for representing actual gene sequences. The data imputation using this method is limited to a simulation of the data to solve a missing data issue in existing datasets for machine learning purpose, and it should not replace the attempt to collect high quality and precise data from the original source if accessible. Using the imputed data for a task that requires precise data, especially the task relates to living being or having an effect on lives, is strongly not advised as it comes with the risk of misinterpretation and incorrect analysis result.

## Conclusion

The ultimate goal of imputation is to fill the missing values to complete a dataset for data analysis and use in automate tasks with machine learning. By filling the missing values, existing methods aim to replicate the missing data considering what is missing. However, as we aim to use the dataset in automate tasks, the dataset will need the process of selecting a subset of relevant features to identify the most informative and discriminative features that contribute the most to the predictive performance or the interpretability of a machine learning model. This research thus focuses on imputation that does not only generate the missing data but also enhances the informativeness and discriminate power of imputed data in the same time.

In this paper, we propose an imputation of missing values towards improvement of accuracy performance for classification in a task of predicting cancer diseases from gene expression. The method is based on missing data imputation which applies Bee algorithm and K-nearest neighborhood with linear regression to generate more impactful values to increase accuracy of prediction. As GINI importance score is utilized in selecting values for imputation, the imputed values reflected on improving a discriminative power in classification tasks instead of replicating the actual values from the original dataset. From evaluation results, the proposed method obtains higher accuracy score than the frequently used imputation methods including K-nearest neighborhood, PCA, LLS, SVD and NLPCA from all applied 3 cancer-identifying gene expression datasets. In comparison of the prediction model from original dataset without missing data, the classification model from imputed datasets yielded 15-25% higher accuracy in class prediction. This result signifies that feature ranking for classification is significantly changed as the imputed data boost a discriminative power, especially in classification.

As the current method is designed to tackle numerical values, we plan to expand it to cover a categorical data type and test it with a dataset containing mixed data type. Furthermore, we will apply the BKL imputation method to synthesize data to limited data source in improving its ability for machine learning tasks. We also plan to apply the BKL imputation method to solve an imbalanced data issue and compare the results against existing oversampling methods.
